# Relationship between medial meniscus extrusion and cartilage measurements in the knee by fully automatic three-dimensional MRI analysis

**DOI:** 10.1186/s12891-020-03768-3

**Published:** 2020-11-12

**Authors:** Hayato Aoki, Nobutake Ozeki, Hisako Katano, Akinobu Hyodo, Yugo Miura, Junpei Matsuda, Kimiko Takanashi, Kenji Suzuki, Jun Masumoto, Noriya Okanouchi, Takeo Fujiwara, Ichiro Sekiya

**Affiliations:** 1 Center for Stem Cell and Regenerative Medicine, Department of Applied Regenerative Medicine, Tokyo Medical and Dental University, 1-5-45 Yushima, Bunkyo-ku, Tokyo, 113-8510 Japan; 2 FUJIFILM Corporation, Tokyo, Japan; 3 Kanagawa Prefectural Government, Kanagawa, Japan; 4 Department of Global Health Promotion, Tokyo Medical and Dental University, Tokyo, Japan

**Keywords:** Cartilage, Meniscus, 3D MRI, U-net, CNN

## Abstract

**Background:**

We developed a fully automatic three-dimensional knee MRI analysis software that can quantify meniscus extrusion and cartilage measurements, including the projected cartilage area ratio (PCAR), which represents the ratio of the subject’s actual cartilage area to their ideal cartilage area. We also collected 3D MRI knee data from 561 volunteers (aged 30–79 years) from the “Kanagawa Knee Study.” Our purposes were to verify the accuracy of the software for automatic cartilage and meniscus segmentation using knee MRI and to examine the relationship between medial meniscus extrusion measurements and cartilage measurements from Kanagawa Knee Study data.

**Methods:**

We constructed a neural network for the software by randomly choosing 10 healthy volunteers and 103 patients with knee pain. We validated the algorithm by randomly selecting 108 of these 113 subjects for training, and determined Dice similarity coefficients from five other subjects. We constructed a neural network using all data (113 subjects) for training. Cartilage thickness, cartilage volume, and PCAR in the medial femoral, lateral femoral, medial tibial, and lateral tibial regions were quantified by using the trained software on Kanagawa Knee Study data and their relationship with subject height was investigated. We also quantified the medial meniscus coverage ratio (MMCR), defined as the ratio of the overlapping area between the medial meniscus area and the medial tibial cartilage area to the medial tibial cartilage area. Finally, we examined the relationship between MMCR and PCAR at middle central medial tibial (mcMT) subregion located in the center of nine subregions in the medial tibial cartilage.

**Results:**

Dice similarity coefficients for cartilage and meniscus were both approximately 0.9. The femoral and tibial cartilage thickness and volume at each region correlated with height, but PCAR did not correlate with height in most settings. PCAR at the mcMT was significantly correlated with MMCR.

**Conclusions:**

Our software showed high segmentation accuracy for the knee cartilage and meniscus. PCAR was more useful than cartilage thickness or volume since it was less affected by height. Relations ips were observed between the medial tibial cartilage measurements and the medial meniscus extrusion measurements in our cross-sectional study.

**Trial registration:**

UMIN, UMIN000032826; 1 September 2018,

## Introduction

Three-dimensional magnetic resonance imaging (3D MRI) analysis is useful for the measurement of cartilage and the meniscus in patients with osteoarthritis (OA) of the knee [1–3]. However, this promising method is not popular at present because segmentation of cartilage and meniscus often requires manual operation or correction, which requires time and effort. These problems have been addressed by the development of automatic segmentation techniques using deep neural networks [4–7]. We have also developed a novel software for automatic extraction of cartilage and meniscus using deep neural networks. One purpose of the present study was to verify the accuracy of the software for automatic cartilage and meniscus segmentation in knee MRI.

**Fig. 1:**
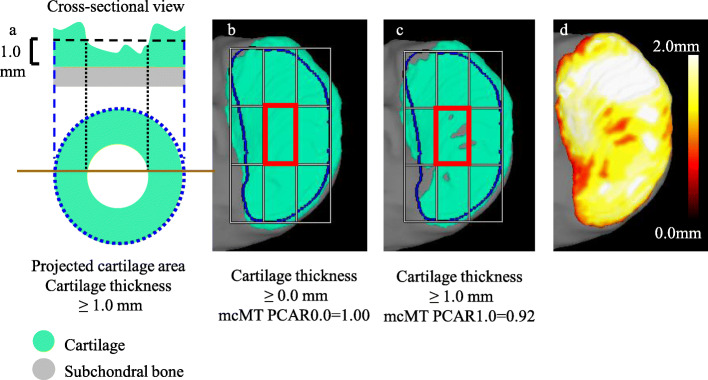
Description of the projected cartilage area and the projected cartilage area ratio (PCAR). **a** Schematic diagram of cross-sectional view of cartilage and the projected cartilage area (cartilage thickness > 1.0 mm). The cross-sectional view is indicated by the brown line in the projected cartilage area. The PCAR is defined as the ratio of the green area to the area enclosed by the blue line (ROI). **b** Practical example of the projected cartilage area (cartilage thickness > 0.0 mm) in the medial tibial cartilage and the PCAR at middle central medial tibia (mcMT) subregion. The ROI of the medial tibial cartilage was defined based on bone morphology and is circled by the blue line. The ROI was divided into nine subregions and the mcMT subregion was surrounded by red square. In this case, mcMT PCAR0.0 was 1.00. **c** Practical example of the projected cartilage area (cartilage thickness > 1.0 mm) in the medial tibial cartilage and the PCAR at mcMT subregion. In this case, mcMT PCAR1.0 was 0.92. **d** Cartilage thickness mapping in the medial tibial cartilage. The color bar shows the cartilage thickness

Measurement of knee cartilage using 3D MRI typically involves the use of cartilage thickness, volume, and thickness maps [1, 8–13]. We recently proposed an additional cartilage measurement, the “projected cartilage area ratio” (PCAR) (Fig. 1), which represents the ratio of a subject’s actual cartilage area to that patient’s ideal cartilage area, defined as a region of interest (ROI) that is predicted from bone morphology. A value of PCAR = 1 means that cartilage covers the ROI entirely, whereas PCAR = 0 means that no cartilage covers the ROI. By adjusting the threshold for cartilage thickness, PCAR can detect subtle changes in cartilage coverage [14]. Furthermore, PCAR, unlike cartilage thickness and volume, may not be affected by body size and may have an advantage when analyzing the cartilage. However, our previous developments did not address the tibial cartilage, and the 3D-reconstructed femoral cartilage was projected directly onto the 2D plane. This resulted in a greater apparent thickness in the 2D projection than the actual thickness due to the slope of the cartilage [14]. To overcome this problem, we have improved the assessment of the 3D-reconstructed femoral cartilage by projecting it cylindrically onto a 2D plane. We also recently developed a PCAR evaluation for tibial cartilage.

The meniscus plays a critical role in shock absorption and is especially important in regulating load-bearing distribution. Meniscus extrusion is one of the strongest risk factors for the progression of OA [15]. Measurements of meniscus extrusion are usually derived from the deviation width of the meniscus in the coronal view of 2D MRI or ultrasound images [16]. However, an extruded meniscus does not always simply displace externally, and an evaluation in only one plane cannot explain the full details of the pathological condition of the extrusion. By contrast, 3D MRI analysis can provide the medial meniscus coverage ratio (MMCR), defined as the ratio of the overlapping area between the medial meniscus area and the medial tibial cartilage area to the medial tibial cartilage area [3, 17–19]. This measurement can be more useful for analyzing the relationship between meniscus extrusion and OA. Therefore, the second purpose of our study was to examine the relationship between MMCR and cartilage measurements, including PCAR, by fully automatic three-dimensional MRI analysis. We performed this analysis by collecting 3D MRI knee data from 561 volunteers, including groups of more than 50 females and 50 males in their 30s, 40s, 50s, 60s, and 70s from the Kanagawa Knee Study.

## Materials and methods

This study was approved by the Medical Research Ethics Committee of Tokyo Medical and Dental University and written informed consent was obtained from all participants. The protocols were enrolled in a database of the National University Hospital Council of Japan (UMIN000031924, UMIN000032826) and disclosed.

### Magnetic resonance imaging (MRI)

**Fig. 2:**
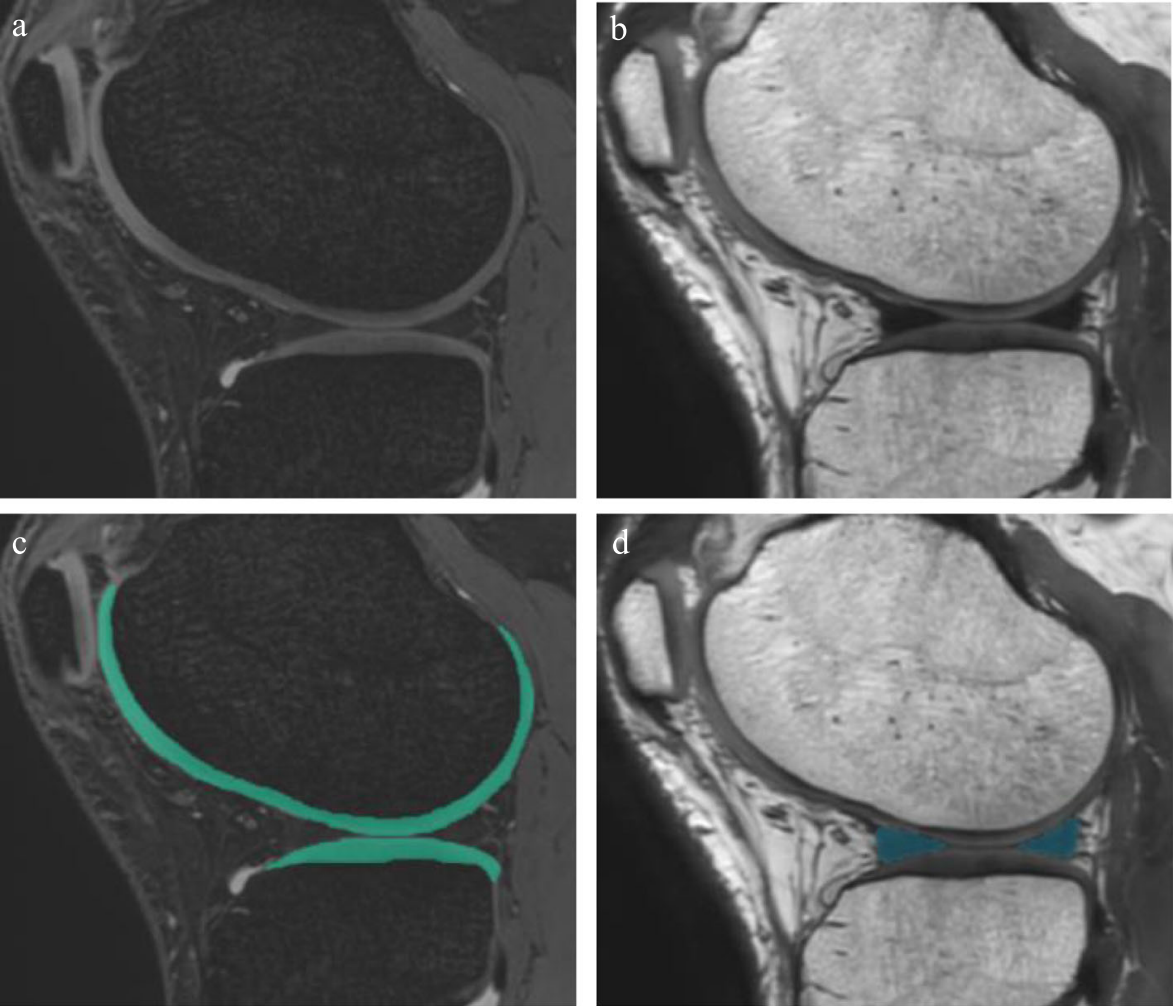
2D MRI of the knee for 3D analyses. **a** Fat-suppressed spoiled gradient echo (SPGR) image. **b** Proton density weighted imaging 3D fast spin echo/ turbo spin echo (PDWI 3D FSE/TSE) image. **c** Femoral and tibial cartilage automatically extracted from SPGR image. The extracted cartilage is shown in green. **d** The meniscus automatically extracted from PDWI 3D FSE/TSE image. The extracted meniscus is shown in blue

Images were collected with a 3.0-T MRI (Achieva 3.0 T TX; Philips) using 16-channel flex coils. The cartilage data were extracted by imaging the sagittal plane of the knee joint using both a fat-suppressed spoiled gradient echo (SPGR) sequence (repetition time, 20 msec; echo time, 1st 7 msec, 2nd 13.8 msec; matrix, 256 × 256; flip angles, 90 deg; slice thickness, 0.3 mm; field of view, 150 mm × 150 mm; Actual Water Fat Shift/ Bandwidth (WFS/BW), 2.002 pix/217.0 Hz; total examination time, 7 min 34 s) (Fig. 2a). The meniscus and bone data were extracted with a proton density weighted imaging 3D fast spin echo/ turbo spin echo (PDWI 3D FSE/TSE) sequence (repetition time, 1000 msec; echo time, 35 msec; matrix, 256 × 256; flip angles, 35 deg; slice thickness, 0.3 mm; field of view, 150 mm × 150 mm; WFS/BW, 0.836 pix/519.4 Hz; total examination time, 7 min 30 s) (Fig. 2b) (Table 1).

**Table 1:** Imaging parameters for the magnetic resonance imaging (MRI) sequences

	SPGR	PDWI 3D FSE/TSE
Repetition time (msec)	20	1000
Echo time (msec)	1st:7	35
	2nd:13.8	
Flip angles (deg)	90	35
Acquisition matrix size	256 × 256	256 × 256
Reconstruction matrix size	512 × 512	512 × 512
No. of sections	320	320
Slice thickness (mm)	0.3	0.3
Field of view (mm × mm)	150 × 150	150 × 150
WFS/BW (pix/ Hz)	2.002/217.0	0.836/519.4
Total examination time	7 min 34 s	7 min 30 s

*SPGR* Fat-suppressed spoiled gradient echo, *PDWI 3D FSE/TSE* Proton density weighted imaging 3D fast spin echo/ turbo spin echo, *WFT/BW* Actual Water Fat Shift/ Bandwidth

### Automatic segmentation algorithm of 3D MRI

**Fig. 3:**
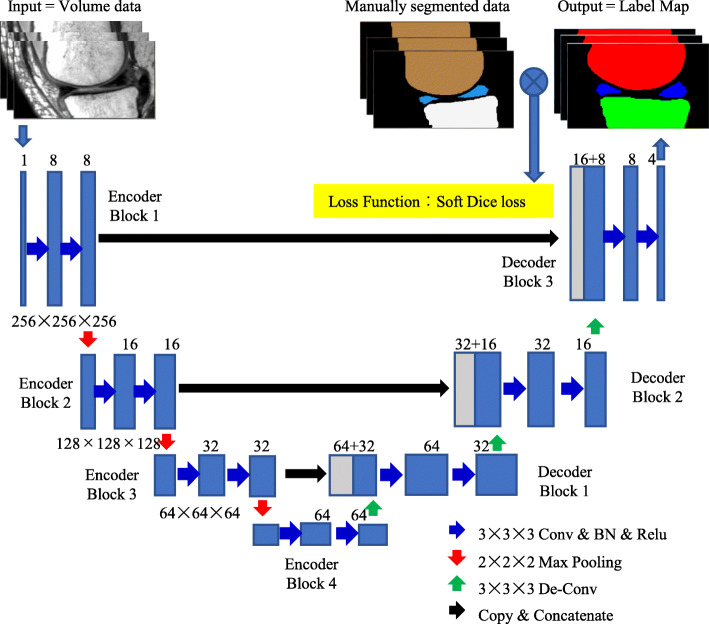
3D convolutional neural network (3D-CNN) algorithm for segmentation of cartilage, meniscus, and bone

A 3D Convolutional Neural Network (3D-CNN) algorithm for segmentation of cartilage (Fig. 2c), meniscus (Fig. 2d), and bone was constructed based on U-Net containing an encoder and a decoder [20] (Fig. 3). The encoder contains four blocks, each consisting of two 3 × 3 × 3 convolution layers, a batch normalization layer, and a rectified linear unit layer. The first 3 blocks also have a max pooling layer with a stride of 2. The decoder contains three blocks; each one had an up-sampling layer, a fusion layer, and two 3 × 3 × 3 de-convolution layers. We used two 3 × 3 × 3 convolutions, instead of a 5 × 5 × 5 convolution, because they can achieve the same reception field with a smaller number of parameters. The inputs were the PDWI 3D FSE/TSE MRI image for meniscus and bone segmentation and the SPGR MRI image for cartilage segmentation. The outputs were probability maps of target regions, including the background region. Two models with the same structure were trained individually on the PDWI 3D FSE/TSE and SPGR MRI image, the former for bone and meniscus segmentation and the latter for cartilage segmentation.

The model was implemented in TensorFlow (https://www.tensorflow.org/). The MRI images were inputted to the CNN, and probability maps of target regions, including the background region were outputted. The ground truth of the target regions and background region were also represented as probability maps with values of 0 or 1. The model was trained by maximizing the dice rate between the probability maps of the ground truth and that outputted by the CNN using the Adam optimizer available in TensorFlow. After the CNN model was trained, the image for segmentation was inputted into the trained model to obtain probability maps of target regions and background region. For each pixel in the image, we found the number of probability maps having maximal probability at the specific pixel and assigned that number as the region label of the pixel to then get a segmentation of the image.

For neural network training, we randomly chose 10 healthy volunteers and 103 patients with knee pain who had visited our hospital between July 7, 2012, and July 24, 2018. These data were manually segmented by two authors (A.H. and H.A.) who had both trained as orthopedic surgeons for 6 years and had experience in the manual correction of over 200 knees. A.H. manually segmented the femoral cartilage and H.A. manually segmented the tibial cartilage and meniscus. These segmentation data were converted by professional engineers (K.S. and J.Mas.) to train the neural network. The network was trained to construct a region of interest (ROI) of the femoral subchondral bone and the medial/lateral tibial plateau by manually segmenting the ROI using a reconstructed 3D knee model.

We ran a validation test for our algorithm by randomly selecting 108 of the 113 subjects were randomly selected for training, and other 5 subjects were used for a validation test by computing the Dice similarly coefficient [21]. Because of small sample size, we performed the validation test three times, selecting 108 different subjects for training and 5 different subjects for each test. After completing three validation tests, the software was trained by all 113 subjects and was then used for the cross-sectional research in this study.

### Kanagawa knee study

The purpose of Kanagawa Knee Study is to clarify the epidemiology and natural history of knee OA, to obtain evidence for the development of diagnosis and treatment, and to identify specific target groups for cartilage and meniscus regenerative medicine for knee OA. The main inclusion criteria are (1) employees of the Kanagawa Prefectural Office, retired employees of the Kanagawa Prefectural Office, or those who work in Kanagawa Prefecture or live in the Tokyo metropolitan area; (2) those who work at a desk for at least 4 h per day or perform similar work during their employment; and (3) those who are able to come to the Tokyo Station area. The main exclusion criteria are those who have (1) a history of surgery on either the left or right knee; (2) a past history of consecutive visits to the hospital for more than 3 months for knee injuries on either the left or right; (3) a history of OA or fractures in either the left or right lower limb (from hip to foot); (4) rheumatoid arthritis or other collagen diseases; and (5) an awareness that they perform strenuous sports on a daily basis, such as full marathons, triathlons, and weightlifting. The main data collected for the study include (1) a questionnaire that covers height, weight, history of knee pain, activity level, Knee Injury and Osteoarthritis Outcome Score (KOOS), and Numerical Rating Scale (NRS); (2) MRI and radiographs of the right knee; and (3) urine output.

We collected 561 datasets including more than 50 females and 50 males per age group (30s, 40s, 50s, 60s, and 70s). The subject size was based on the study budget. We announced recruitment of these subjects at the Kanagawa Prefectural Government between September 1, 2018 and August 30, 2019. Participants joined our study voluntarily. For the first data set, we collected questionnaires, knee radiographs, and MRIs between November 3, 2018, and September 28, 2019, at the AIC Yaesu clinic of Tokyo. We plan to collect these data twice, with an interval of 1 year. A second data set is currently being collected. “Only the first data set was analyzed in this paper.”

### Cartilage measurements

**Fig. 4:**
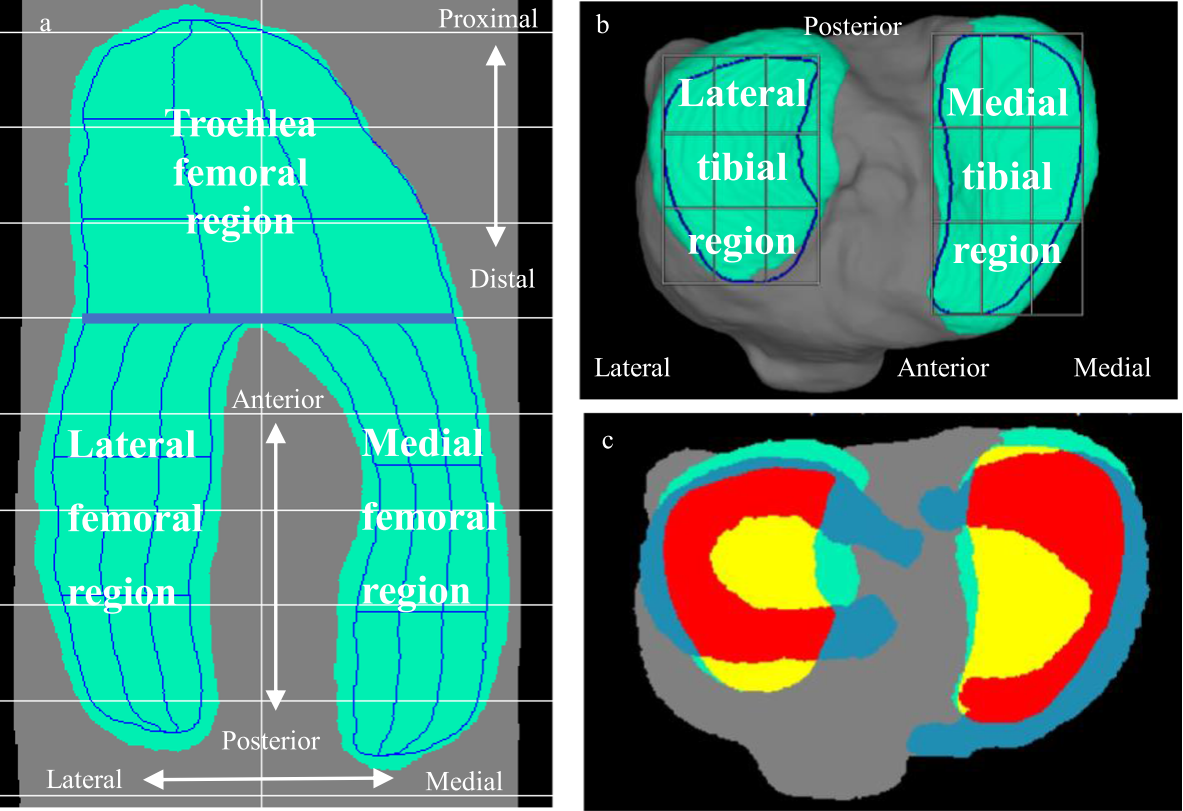
Cartilage regions and the meniscus coverage ratio (MCR). **a** Three regions of femoral cartilage. The green area is the femoral cartilage. The ROI of the femoral cartilage was defined based on bone morphology and surrounded by the blue outer line. The ROI was divided into 27 subregions, indicated by blue lines, and curves were automatically determined by CNN. **b** Two subregions of the tibial cartilage. **c** Schematic diagram of the MMCR. For the medial tibial articular surface, the cartilage area, ROI area, and the medial meniscus area and their overlap area are shown in different colors. The red area shows the overlap between the ROI area and the medial meniscus area. The yellow area shows the ROI area that does not overlap with other areas. The green area shows the cartilage area that does not overlap with the other areas. The blue area shows the medial meniscus area that does not overlap with the ROI area. A similar explanation can be given for the lateral meniscus

The software we used for MRI analyses was a 3D image analysis system volume analyzer (SYNAPSE 3D, Collaborative version, FUJIFILM Corporation, Tokyo, Japan). We quantified the cartilage by projecting the femoral cartilage cylindrically and dividing it into three regions inside the ROI based on the femoral bone (Fig. 4a). The tibial cartilage was vertically projected and divided into two areas inside the ROI at the medial tibia and lateral tibial plateau (Fig. 4b). Each area was automatically divided into 3 × 3 subregions at equal intervals [22].

Our software automatically computed the average cartilage thickness (ThC), cartilage volume (VC), and projected cartilage area ratio (PCAR) in each region and subregion. Our software could also display the cartilage thickness mapping (Fig. 1d). PCAR represented the ratio of the projected cartilage area to the total area of the ROI. We evaluated PCAR for the threshold of cartilage thicknesses at > 0.0 mm, > 0.5 mm, > 1.0 mm, and > 1.5 mm. The PCAR values for the thresholds of cartilage thicknesses at each of these measurements were designated PCAR0.0, PCAR0.5, PCAR1.0, and PCAR1.5, respectively.

#### Meniscus extrusion measurements

The medial meniscus extrusion (MME) area, MME volume, and medial meniscus coverage ratio (MMCR) were automatically computed. The MME area was defined as the area of the medial meniscus that did not overlap with the area of ROI of the medial tibial plateau (Fig. 4c). The MME volume was defined as the 3D measurement of the MM segment of the MME area. MMCR was defined as the ratio of the overlapping area of the medial meniscus and the area of ROI of the medial tibial plateau (Fig. 4c).

### Statistical analysis

We evaluated the accuracy of automatic segmentation by calculating the Dice similarity coefficient (DSC) between manual segmentation and automatic segmentation [21]. For each validation test, the DSC was computed for five test subjects at the femoral bone, tibial bone, femoral cartilage, tibial cartilage, medial/lateral meniscus, femoral subchondral bone ROI, and medial/lateral tibia plateau ROI. After the three validation tests, we calculated the mean DSC of each test.

We evaluated the correlation between each quantitative value and other quantitative value using Spearman’s rank correlation test. All statistical analyses were performed using JMP® 14 (SAS Institute Inc., Cary, NC, USA). *P* values < 0.05 were considered statistically significant.

## Results

### Segmentation accuracy

The mean DSC of all tests combined was 0.985 for the femoral bone, 0.980 for the tibial bone, 0.911 for the femoral cartilage, 0.892 for the tibial cartilage, 0.916 for the medial meniscus, 0.891 for the lateral meniscus, 0.905 for the ROI of the femoral subchondral bone, and 0.888 for the ROI of the medial/lateral tibia plateau (Table 2).

**Table 2:** Automatic segmentation accuracy

	DSC (average ± SD)			
Subregions	1st test	2nd test	3rd test	Total
Femoral bone	0.985 ± 0.004	0.985 ± 0.007	0.983 ± 0.004	0.985 ± 0.005
Tibial bone	0.981 ± 0.008	0.978 ± 0.013	0.983 ± 0.002	0.980 ± 0.009
Femoral cartilage	0.905 ± 0.024	0.913 ± 0.034	0.916 ± 0.013	0.911 ± 0.024
Tibial cartilage	0.893 ± 0.072	0.868 ± 0.119	0.914 ± 0.009	0.892 ± 0.072
Medial meniscus	0.921 ± 0.012	0.906 ± 0.020	0.922 ± 0.014	0.916 ± 0.016
Lateral meniscus	0.893 ± 0.038	0.905 ± 0.033	0.875 ± 0.053	0.891 ± 0.041
ROI of femoral subchondral bone	0.905 ± 0.016	0.904 ± 0.019	0.906 ± 0.017	0.905 ± 0.016
ROI of medial/lateral tibia plateau	0.889 ± 0.036	0.884 ± 0.055	0.892 ± 0.022	0.888 ± 0.037

*DSC* Dice similarly coefficient, *ROI* Region of interest

*DSC averages and SDs were calculated for the first to third tests with 5 subject data, for a total of 15 subject data

### Characteristics of study subjects in Kanagawa knee study

This study included 561 subjects: 277 females and 284 males (Table 3). The overall average age of the subjects was 53.7 ± 13.9 years for females and 55.2 ± 11.0 years for males. The body mass index was 22.5 ± 3.0 (kg/cm^2^) for females and 23.6 ± 3.2 (kg/cm^2^) for males. The rate of grade 3 or 4 on the Kellgren–Lawrence OA scale was 5.4% for females and 1.4% for males.

**Table 3:** Characteristics of the study subjects

Characteristic	Female	Male
Total	*n* = 277	*n* = 284
Age	53.7 ± 13.9	55.2 ± 11.0
30–39 years	*n* = 56	*n* = 52
40–49 years	*n* = 61	*n* = 62
50–59 years	*n* = 53	*n* = 50
60–69 years	*n* = 55	*n* = 57
70–79 years	n = 52	*n* = 63
Height: means ± SD (cm)	158.6 ± 7.1	168.4 ± 5.5
Weight: means ± SD (kg)	56.7 ± 9.2	66.9 ± 11.2
Body mass index: means ±SD (kg/cm^2^)	22.5 ± 3.0	23.6 ± 3.2
Kellgren–Lawrence OA scale KL0/1/2/3/4	231/23/8/6/9	266/9/5/3/1

*OA* Osteoarthritis, *SD* Standard deviation

### Correlation between cartilage measurements and body size

The thickness and volume of the femoral and tibial cartilage in each region were correlated with height in both genders (Table 4). By contrast, the medial femoral (MF) PCAR, lateral femoral (LF) PCAR, medial tibial (MT) PCAR, and lateral tibial (LT) PCAR0.0–1.5 were not correlated with height. Even if a correlation was present, the rs values were less than 0.2 in both genders. No correlations were noted between cartilage measurements (CV, ThC, and PCAR) and weight, or between cartilage measurements and BMI (data not shown).

**Table 4:** Correlation quantification of cartilage with height

Quantification of each cartilage	rs	*p* value
Female		
F PCAR0.0	0.07	0.23
F PCAR0.5	0.07	0.21
F PCAR1.0	0.10	0.09
F PCAR1.5	0.12	0.06
F.ThC	0.18	0.003
F.VC	0.39	<.0001
LT PCAR0.0	0.08	0.21
LT PCAR0.5	0.07	0.25
LT PCAR1.0	0.08	0.18
LT PCAR1.5	0.13	0.04
LT.ThC	0.19	0.001
LT.VC	0.36	<.0001
MT PCAR0.0	− 0.03	0.58
MT PCAR0.5	− 0.04	0.54
MT PCAR1.0	0.01	0.82
MT PCAR1.5	0.11	0.06
MT.ThC	0.14	0.02
MT.VC	0.32	<.0001
Male		
F PCAR0.0	0.04	0.48
F PCAR0.5	0.04	0.52
F PCAR1.0	0.09	0.13
F PCAR1.5	0.16	0.01
F.ThC	0.34	<.0001
F.VC	0.49	<.0001
LT PCAR0.0	0.15	0.01
LT PCAR0.5	0.15	0.01
LT PCAR1.0	0.14	0.02
LT PCAR1.5	0.15	0.01
LT.ThC	0.25	<.0001
LT.VC	0.43	<.0001
MT PCAR0.0	0.01	0.81
MT PCAR0.5	0.01	0.84
MT PCAR1.0	0.13	0.03
MT PCAR1.5	0.16	0.01
MT.ThC	0.20	<.0001
MT.VC	0.38	<.0001

*F* Femur, *LT* Lateral tibia, *MT* Medial tibia, *PCAR* Projected cartilage area ratio, *ThC* Average cartilage thickness, *VC* Cartilage volume

### Correlation between cartilage at mcMT and MMCR

We focused on the mcMT subregion (Fig. 1 b, c) to examine the relationship between cartilage measurements and MME area, MME volume and MMCR in nine subregions in each of the five cartilage subregions (Fig. 4 a, b), since that subregion was considered to be more representative than the other subregions. We also focused on the PCAR among the cartilage measurements that included cartilage thickness and volume since PCAR values were cartilage measurements that were not affected by height. In females, mcMT PCAR0.0, 0.5, and 1.0 were significantly correlated with the MME area, MME volume, and MMCR, while mcMT PCAR1.5 was correlated with MMCR (Table 5). In males, mcMT PCAR0.0, 0.5, and 1.0 were significantly correlated with the MME area, MME volume, and MMCR, with the exception of the correlation between PCAR0.5 and MMCR. However, their absolute values of rs were less than 0.2.

**Table 5:** Correlation of PCAR at mcMT and MMCR

	rs (*p* value)			
	mcMT PCAR0.0	mcMT PCAR0.5	mcMT PCAR1.0	mcMT PCAR1.5
Female				
MME Area	−0.32 (< 0.0001)	−0.32 (< 0.0001)	−0.19 (0.0017)	−0.05 (0.38)
MME Volume	−0.32 (< 0.0001)	− 0.32 (< 0.0001)	− 0.18 (0.0022)	− 0.02 (0.67)
MMCR	0.30 (< 0.0001)	0.30 (< 0.0001)	0.25 (< 0.0001)	0.28 (< 0.0001)
Male				
MME Area	−0.18 (0.0029)	−0.17 (0.0036)	−0.17 (0.0036)	−0.01 (0.82)
MME Volume	−0.18 (0.0029)	−0.18 (0.0022)	−0.18 (0.0029)	−0.01 (0.90)
MMCR	0.17 (0.0033)	0.11 (0.057)	0.13 (0.033)	0.04 (0.54)

*mcMT* Medial central medial tibia, *MME* Medial meniscus extrusion, *MMCR* Medial meniscus coverage ratio, *PCAR* Projected cartilage area ratio

## Discussion

This software developed for 3D analysis of knee MRI has eight main functions: (1) it automatically extracts bones, cartilage, and meniscus; (2) it constructs bones, cartilage, and meniscus in three dimensions; (3) it projects the tibial cartilage in a plane; (4) it projects the femoral cartilage cylindrically in a plane; (5) it sets the ROI of three adjacent cartilage regions for the femoral cartilage and two separate cartilage regions for the tibial cartilage based on the morphology of the bones; (6) it divides each region into nine subregions; (7) it quantifies cartilage thickness, volume and PCAR; and (8) it quantifies the meniscus coverage ratio. This is the first report in which we have used this software to analyze data from an epidemiological study.

For quantitative knee cartilage morphometry, many reports use one sequence, the 3D dual-echo in steady state (DESS), which is recommended by OAI [23]. We used two types of sequences; the SPGR is superior for discrimination of cartilage, whereas the PDWI 3D FSE/TSE is useful for defining the meniscus. Although the use of the two sequences increases the imaging time, the total time for the two sequences can be kept within 20 min.

The thickness and volume of the femoral and tibial cartilage in each region were correlated with height. Some reports have described the usefulness of cartilage thickness and volume as cartilage measurements [1, 8–13]; however, our results indicate that these measurements need adjustment for each individual’s height for use in a cross-sectional study. By contrast, MF PCAR0.0–1.5 and MT PCAR0.0–1.5 were not correlated with height. The PCARs are more useful as cartilage measurements than are cartilage thickness and volume because the PCAR does not need to be corrected for height.

MME is a known risk factor for the occurrence of OA [15]. MME is usually quantified by measuring the distance between the medial meniscus edge and the tibial plateau edge using 2D MRI or coronal ultrasound [16]. We automatically calculated the MME area, MME volume, and MMCR. The MME area was defined as the area of the medial meniscus that did not overlap with the area of ROI of the medial tibial plateau. Our definition of MME is unique in that it reflects the extrusion in each direction, including anterior and posterior extrusions and medial extrusion. However, a limitation of our measurements is that the MME area included the anterior and posterior roots of MM, as well as MM between the area of ROI of the medial tibial plateau and the medial margin of the tibial bone. MMCR is a measurement similar to the percentage of the tibial plateau area covered by the meniscus, which has already been proposed by Roth et al. [19], and is useful to overcome this limitation. We showed relationships between the medial tibial cartilage and medial meniscus extrusion measurements in our cross-sectional study.

We had other three limitations. First, the software was specific to the training cohort, and the performance was not the same to the different cohort. Second, we could not calculate intra-rater reliability for segmentation accuracy because one author manually segmented femoral cartilage and another author manually segmented tibial cartilage and meniscus. Third, the composition of the subjects in Kanagawa Knee Study did not match that of the general population and this selection bias cannot be ignored.

## Conclusion

Our software showed high segmentation accuracy for the knee cartilage and meniscus. PCAR was useful as a cartilage measurement since it was less affected by height than were the cartilage thickness and volume. Relationships were observed between the medial tibial cartilage measurements and medial meniscus extrusion measurements in our cross-sectional study.

## Data Availability

The datasets used and/or analyzed during the current study are available from the corresponding author on reasonable request.

## Abbreviations

3D MRI: Three-dimensional magnetic resonance imaging
PCAR: Projected cartilage area ratio
ThC: Cartilage average thickness
VC: Cartilage volume
ROI: Region of interest
ME: Meniscus extrusion
MME: Medial meniscus extrusion
MCR: Meniscus coverage ratio
MMCR: Medial meniscus coverage ratio
SPGR: Spoiled gradient echo
PDWI: Proton density weighted imaging
MF: Medial femoral condyle
LF: Lateral femoral condyle
MT: Medial tibia
LT: Lateral tibia
DSC: Dice similarity coefficient
mc: Medial central subregion
